# Association between genomic features and toxic metal(loid) accumulation in left-sided and right-sided colorectal cancer

**DOI:** 10.3389/fonc.2025.1584424

**Published:** 2025-07-04

**Authors:** Shoujiang Yu, Min Fu, Yurong Wang, Ling Lin, Shushen Ji, Feng Du, Li Song, Mengzhu Wang, Yufeng Zhai, Jiangman Zhao, Min Huang

**Affiliations:** ^1^ Ward I of Department of General Surgery, the First People’s Hospital of Pingdingshan, the First Hospital Affiliated to Pingdingshan University, Pingdignshan, Henan, China; ^2^ Department of Oncology, Nanyang Second People’s Hospital, Nanyang, Henan, China; ^3^ Department of Clinical Laboratory, Nanjing Jiangbei Hospital, Nanjing, China; ^4^ Shanghai Biotecan Medical Laboratory Co., Ltd., Shanghai Zhangjiang Institute of Medical Innovation, Shanghai, China; ^5^ Department of Respiration, Huanghe Sanmenxia Hospital Affiliated to Henan University of Science and Technology, Sanmenxia, China

**Keywords:** colorectal cancer, next-generation sequencing, metal(loid), microsatellite instability, anatomical location

## Abstract

**Background:**

Colorectal cancer (CRC) is a high heterogenous disease of genetic variations, which was influenced by tumor anatomic location and toxic metal(loid) accumulation. Current study aims to investigate genomic heterogeneity of CRC influenced by toxic metal(loid) accumulation based on tumor anatomic location.

**Methods:**

In this study, a total of 94 patients with CRC were recruited including 69 left-sided tumors and 35-right sided tumors. The genomic mutation landscape and microsatellite instability (MSI) of tumors were analyzed. The blood metal(loid) element levels were tested by inductively coupled plasma emission spectrometry (ICP-MS).

**Results:**

A total of 642 somatic variations across 24 genes were identified in 94 CRC patients. The most frequently mutated genes were *TP53* (n=83%), followed by *APC* (n=67%), *KRAS* (52%), *EGFR* (41%) and *PIK3CA* (33%). The mutated frequency of *TP53* (88.4% vs 68.0%, *P*=0.02) and *APC* (75.4% vs 44.0%, *P*=0.004) in left-sided tumors were significantly higher than that of right-sided tumors. Blood Hg concentration was significantly and positively correlated with numbers of variations per tumor sample (r=0.237, *P*=0.021). Blood As (*r*= -0.207, *P*=0.046), Sr (*r*= -0.256, *P*=0.013) and Ba (*r*= -0.274, *P*=0.08) level of patients with MSS tumor was significantly higher than that of patients with MSI tumor. Cd level of patients with tumor in left side was significantly lower than that in right side (*P*=0.028).

**Conclusions:**

This study presented the comprehensive genomic landscape of 94 CRC patients according to tumor anatomic location. The blood toxic metal(loid) accumulation may have potential influence on genomic features.

## 1 Introduction

Colorectal cancer (CRC) ranks as the third most diagnosed cancer and the second leading cause of cancer-related deaths, according to the latest estimates from International Agency for Research on Cancer, IARC) of World Health Organization (WHO) (1). Despite advancements in screening methods, such as colonoscopy and fecal occult blood testing, and therapeutic interventions including surgery, chemotherapy, targeted therapies, and immunotherapy, CRC remains a formidable challenge due to its heterogeneous nature, late diagnosis in many cases, and the development of resistance to treatment. Therefore, there is a pressing need for novel insights into the molecular mechanisms underlying CRC initiation, progression, and metastasis, as well as the identification of biomarkers for early detection and therapeutic targets for personalized medicine.

High-throughput sequencing technologies have revolutionized the field of CRC research by enabling comprehensive characterization of the genetic and epigenetic alterations underlying CRC. Based on these molecular profiles, CRC has been classified into distinct subtypes with unique clinical, pathological, and therapeutic implications. For instance, the Consensus Molecular Subtypes (CMS) classification system, proposed by Guinney et al. (2) categorizes CRC into four major subtypes: CMS1 (MSI immune), CMS2 (canonical), CMS3 (metabolic), and CMS4 (mesenchymal). This classification system has been validated in multiple independent cohorts and has shown to correlate with patient outcomes and response to therapy. Other molecular subtyping systems have also been proposed, each with its unique set of biomarkers and clinical implications. For example, the Laetitia Marisa et al. have identified six consensus molecular subtypes (C1-C6) (3), which are defined by distinct patterns of gene expression, mutational signatures, and epigenetics, providing further insights into CRC heterogeneity.

Some researchers have highlighted the role of environmental factors, particularly heavy metal exposure, in modulating CRC risk and outcomes. Heavy metals, such as arsenic, cadmium, and lead, are naturally occurring or anthropogenically introduced into the environment and can accumulate in the human body through dietary intake, inhalation, or dermal absorption (4). Accumulation of these metals has been linked to various health issues, including cancer. For instance, arsenic exposure has been associated with increased CRC risk (5, 6). Similarly, cadmium exposure has been implicated in cancer development, potentially through the induction of oxidative stress and genetic alterations (7). Exposure to toxic metal(loid) elements has been reported to can potentially disrupt genomic stability in cancer-related genes (8), such as lung adenocarcinoma and CRC. Furthermore, toxic metal(loid) elements may also modulate the expression of genes involved in DNA repair and apoptosis, further exacerbating genetic instability in CRC (9).

In addition to the influence of environmental factors, CRC exhibits distinct clinical and molecular features based on the location within the colon. Left-sided CRCs and right-sided CRCs have been shown to differ in their epidemiology, clinical presentation, response to therapy, and genetic profiles. Right-sided CRCs are more frequently associated with mutations in *KRAS*, whereas left-sided CRCs tend to harbor mutations in *TP53* (10). These differences underscore the importance of considering tumor location when developing diagnostic, prognostic, and therapeutic strategies for CRC patients.

Given the complexity of CRC and the multitude of factors that contribute to its development and progression, there is a critical need for continued research to better understand the interplay between environmental exposures, such as heavy metal accumulation, and the genetic alterations underlying CRC. Our study aims to contribute to this effort by investigating the specific impact of toxic metal(loid) accumulation on CRC gene mutations, with a particular focus on potential differences between left-sided and right-sided CRCs.

## 2 Methods

### 2.1 Study participants

This is an observational, cohort study. A total of 94 newly diagnosed, primary and pathologically confirmed CRC patients were recruited from The First People’s Hospital of Pingdingshan between May 2020 and October 2023. Inclusion criteria included: newly diagnosed and primary CRC, confirmed by pathological diagnosis, patient informed consent. Exclusion criteria included pregnant women, have undergone anti-cancer treatment such as neoadjuvant chemo-radiotherapy, failure to obtain informed consent, and other underlying disease unsuitable for participant in this study.

### 2.2 Clinical information and sample collection

**Figure 1:**
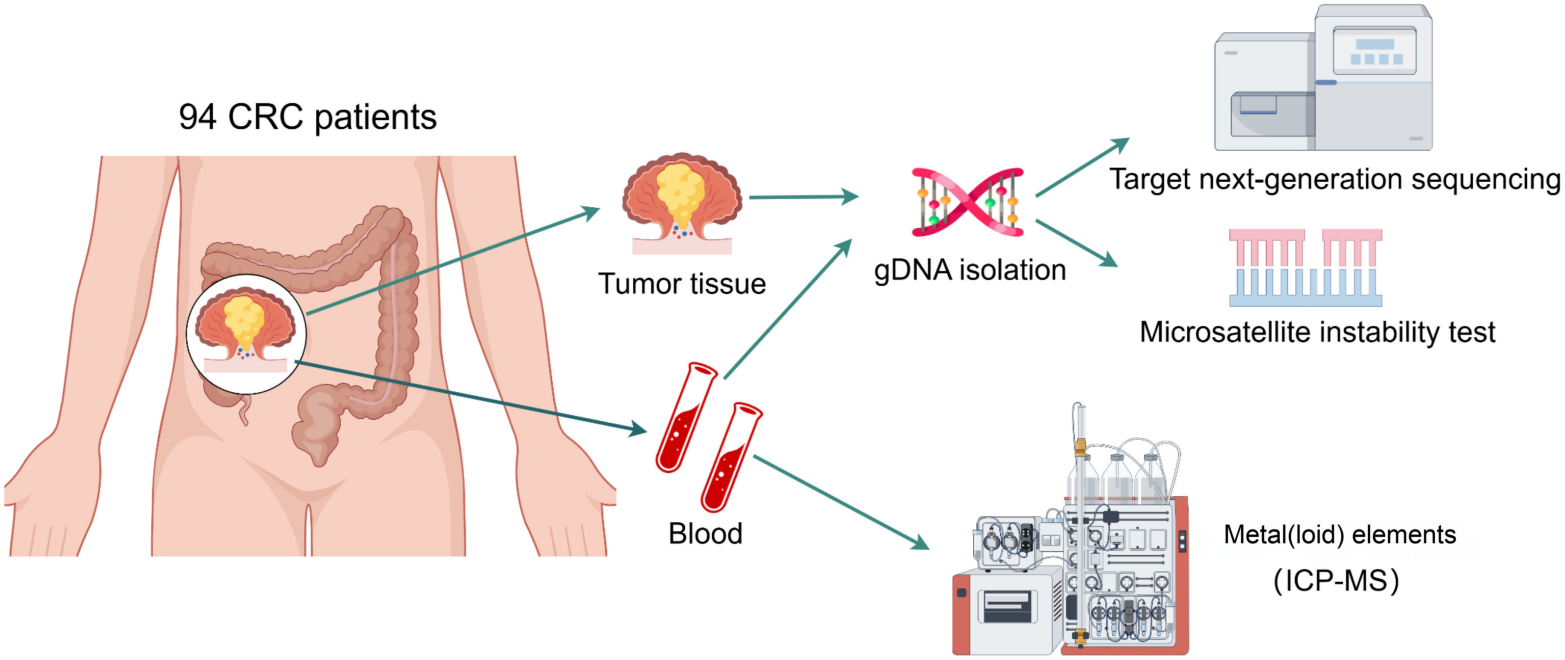
Workflow of study design (poltted by Figdraw 2.0).

Tumor tissues from CRC patients during surgery and peripheral blood were collected for microsatellite instability and target next-generation sequencing (NGS) of 95 cancer-related genes (**Supplementary Table 1**). In addition, additional 2 peripheral blood was collected before any treatment by heparin sodium tube for 18 metal(loid) elements detection. Basic information and clinical diagnostic and treatment information of CRC patients, including gender, age, height, weight, smoking and alcohol history, medical history of underlying diseases, tumor stage, pathological classification, differentiation degree, tumor location, size, tumor biomarkers et al, were collected through electronic medical record systems and survey questionnaires. After hospital discharge, patients underwent regular follow-up evaluations. Recurrence and death events were recorded. Progression-free survival (PFS) was calculated from the date of surgery to the first documented recurrence, and overall survival (OS) was measured from the date of diagnosis to death or the last follow-up time. The study design was shown in **Figure 1**.

### 2.3 Metal(loid) element detection

In this study, eighteen toxic metal(loid) elements were measured, including vanadium (V), chromium (Cr), manganese (Mn), cobalt (Co), nickel (Ni), copper (Cu), zinc (Zn), gallium (Ga), arsenic (As), selenium (Se), strontium (Sr), cadmium (Cd), tin (Sn), antimony (Sb), barium (Ba), mercury (Hg), thallium (Tl), and lead (Pb). The elements were systematically selected based on the following criteria: (1) toxicological relevance, and (2) carcinogenic potential. Metal elements were classified as heavy metals if they met the density threshold(>4.5 g/cm³) and exhibited documented toxicity or carcinogenicity, as per the International Agency for Research on Cancer (IARC) classifications (Group 1: carcinogenic; Group 2A/B: probable/possible carcinogens). Essential micronutrients such as iron (Fe) were excluded due to its essential role. Arsenic (As), though chemically categorized as a metalloid, was included as a “heavy metal” in this analysis due to its high density, environmental persistence, and IARC Group 1 carcinogenicity designation. Selenium (Se) was incorporated to investigate its dual role as an essential nutrient and potential toxin at supraphysiological doses, given emerging evidence of dose-dependent impacts on colorectal carcinogenesis.

It was conducted using an inductively coupled plasma mass spectrometry Agilent 7800 ICP-MS (Agilent Technologies, USA) followed by the manufacturer’s instructions. The experimentation was conducted at ambient temperature, with the samples undergoing a pretreatment involving a 10-fold dilution process by incorporating 0.5 ml sample into a solution consisting of 0.5% nitric acid and 0.1% Triton X-100. Subsequently, the instrument’s operational parameters were meticulously calibrated as follows: a cooling gas flow rate set at 12.5 L/min, an auxiliary gas flow rate adjusted to 0.7 L/min, a nebulizer gas flow rate maintained at 0.92 L/min, a peristaltic pump speed regulated to 20 r/min, a nebulizer temperature precisely controlled at 3°C, a CCT (8% hydrogen in helium) gas flow rate of 3.75 ml/min, and the utilization of a nickel sampler cone with a diameter of 1.1 mm.

### 2.4 Microsatellite instability test

DNA extraction and quality control refer to the published literature of our team (11). The method for microsatellite instability testing was referenced from previously published research (12) by our team. In brief, MSI status was conducted utilizing a panel of five microsatellite markers, comprising two mononucleotide repeats (BAT25, BAT26) and three dinucleotide repeats (D2S123, D5S346, D17S250), through PCR amplification. Subsequently, PCR products were subjected to capillary electrophoresis using the ABI 3730XL DNA Analyzer (ABI, USA). MSI-H status was assigned when at least two of the five markers exhibited instability in tumor DNA. MSI-L status was designated when one MSI marker displayed instability, while tumors were classified as MSS if no instability was observed in the tumor DNA.

### 2.5 Library preparation and sequencing

Firstly, 300 nanograms of genomic DNA per sample were fragmented using a Covaris E220 focused ultrasonicator (Covaris, LLC.) to obtain DNA fragments of 150–200 base pairs. Subsequently, 10–100 nanograms of DNA fragments were employed for library construction using the KAPA library preparation kit (Kapa Biosystems Inc.; Roche Diagnostics). Thirdly, the NGS libraries were captured using the xGen Lockdown Probe pool (Integrated DNA Technologies, Inc.), and the captured DNA fragments were amplified using 1X KAPA HiFi Hot Start Ready Mix (Kapa Biosystems Inc.; Roche Diagnostics). Finally, the Illumina NextSeq CN500 platform with a medium flux chip (NextSeq CN500 Mid Output v2 kit; Illumina Inc.) was utilized for cluster generation.

### 2.6 Bioinformatics analysis

To obtain clean data, the process involved filtering out adapter, low-quality reads, and reads with proportion of N > 10% by fastqc software (v.0.11.8). Subsequently, these filtered reads underwent alignment to the human genome, specifically the UCSC hg19 reference (University of California Santa Cruz, hg19), utilizing the Burrows-Wheeler Aligner software version 0.7.17. Following alignment, the Picard and Genome Analysis Toolkit version 4 (GATK4 v.4.2.6.1) methodologies were employed to eliminate duplicates, perform local realignment, and recalibrate base quality scores, simultaneously generating quality statistics. Ultimately, vardict software version 1.8.0 (13) was utilized for variant detection, a total of 18,539 single nucleotide variations (SNVs) and insertions/deletions (InDels) were detected across the 94 samples.

After applying the five filtering steps, a total of 642 confident somatic mutations were selected from the 94 samples for subsequent analysis: (1) Filter for exonic and splicing mutations, excluding synonymous mutations; (2) Exclude variant sites with supporting reads less than 10; (3) Select variants with an allele frequency less than 0.001 or not recorded in 1000 Genomes databases (1000 Genomes Project Consortium; https://www.internationalgenome.org/), or variants with an allele frequency greater than 0.001 but less than 0.1 and recorded in the COSMIC database (http://cancer.sanger.ac.uk/cosmic); (4) Filter for variants with an allele frequency less than 0.01 or not recorded in the ExAC database (the Exome Aggregation Consortium); (5) Select variants predicted to be harmful by at least one of the following predictive tools: Polyphen2_HDIV_pred, Polyphen2_HVAR_pred, or MutationTaster_Pred.

### 2.7 Statistical analysis

The mutation landscape across 94 patients, including SNVs, InDels and clinical features, were created by ComplexHeatmap package in R project (14). The custom mutation distribution of proteins in hotspot genes were visualized by maftools package in R (15). Mutually exclusive and co-occurrence genes were identified by maftools package. Ggplot2 package was used to plot, and the nonparametric Mann–Whitney U test was used to test for significant difference of medians between two populations. Progression-free survival (PFS) was analyzed using the Kaplan–Meier method, and survival distributions between subgroups were compared with the log-rank test. *P* value less than 0.05 was defined significance.

## 3 Results

### 3.1 Clinical characteristics of CRC patients

The clinical characteristics of 94 patients diagnosed with CRC are summarized in **Table 1**. The median age at diagnosis was 63 years (range, 29 to 87), and 55.3% of the patients were male. A family history of cancer was reported in 3.2% of patients (n=3), smoking history in 20.2%, and drinking history in 21.3% of patients. According to the American Joint Committee on Cancer (AJCC) staging system, 6.4% of patients belonged to stage I, 47.9% to stage II, and 45.7% to stage III. Of the total cohort, 47.9% of patients had colon cancer, while the remaining 52.1% were diagnosed with rectal cancer.

**Table 1:** Clinical characteristics of 94 CRC patients according to tumor anatomic location.

Clinical characteristics	Total (n=94)	Percentage	Left side (n=69)	Right side (n=35)	P value
Gender					0.072
Male	52	55.3%	42	10	
Female	42	44.7%	27	15	
Age/ median (range)	63 (29-87)				0.936
≤60	42	44.7%	31	11	
>60	52	55.3%	38	14	
BMI					0.998
<18.5	8	8.5%	6	2	
18.5-24	41	43.6%	30	3	
24-28	33	35.1%	24	11	
≥28	12	12.8%	9	3	
Smoking history					0.383
Yes	19	20.2%	16	3	
No	75	79.8%	53	22	
Drinking history					0.698
Yes	20	21.3%	14	6	
No	74	78.7%	55	19	
Family history of cancer					1.000
Yes	3	3.2%	2	1	
No	91	96.8%	67	24	
Stage					0.119
I	6	6.4%	3	3	
II	45	47.9%	37	8	
III	43	45.7%	29	14	
Microsatellite instability					0.286
MSS	80	85.1%	61	19	
MSI-L	8	8.5%	5	3	
MSI-H	6	6.4%	3	3	

### 3.2 Genomic alteration landscape in the cohort

**Figure 2:**
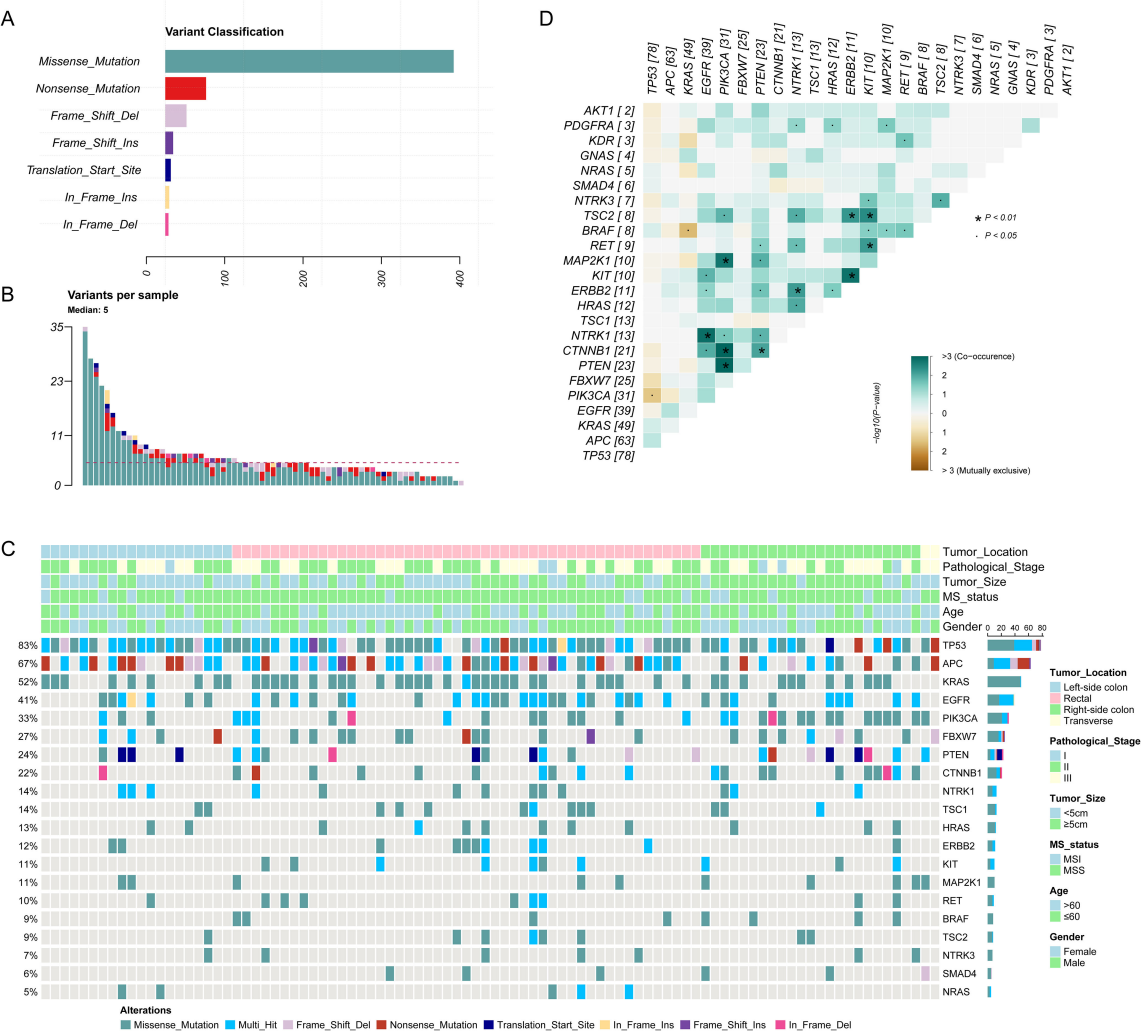
The genomic landscape of 94 colorectal cancer (CRC) patients. **(A)** The distribution of different variant classification; **(B)** The number of variants per sample; **(C)** The genomic landscape of 94 CRC patients according to clinical features. **(D)** Patterns of co-occurrence and mutual exclusivity genes.

The mean depth of clean data was 927 coverage, with average 90% Q30 high-quality reads. In total, 18,539 single nucleotide variations (SNVs) and insertions/deletions (InDels) were detected across the 94 samples. After stringent filtering, a total of 642 confident somatic mutations were selected from the 94 samples for subsequent analysis, including 501 missense mutations, 69 nonsense mutations, 37 frameshift deletions, 12 frameshift insertions, 11 translation start sites, 7 inframeshift deletions and 5 inframeshift insertions (**Figure 2A**). Among the 94 samples, the median number of variants per sample is 5, with range of 1 to 35 (**Figure 2B**).

**Figure 3:**
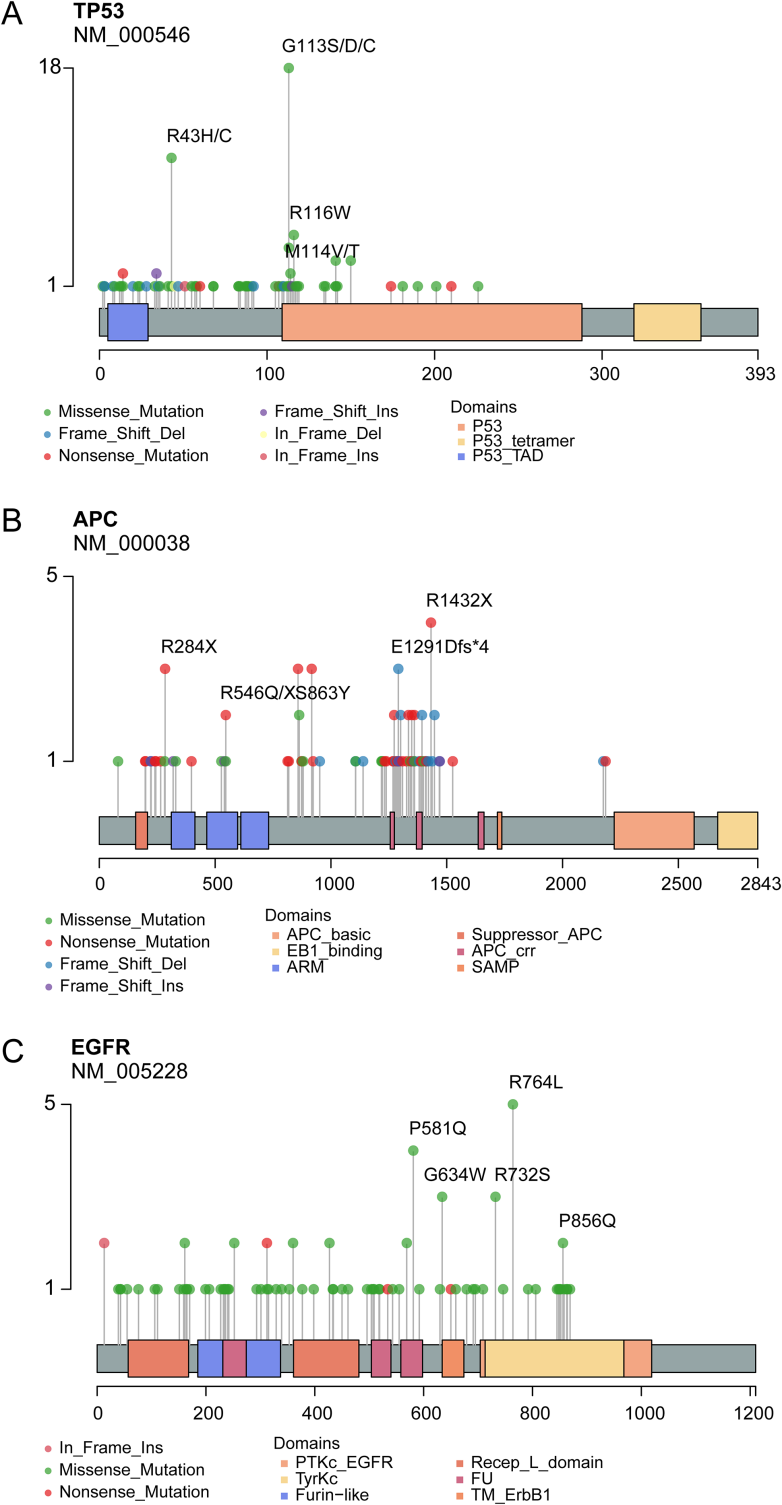
Lollipop plot show the distribution of identified hotspots in *TP53* **(A)**, *APC* **(B)** and *KRAS* **(C)** protein.

The mutational landscape of 20 recurrently somatic mutated genes in this CRC cohort were presented in **Figure 2C**. The most frequently mutated genes were *TP53* (n=83%), followed by *APC* (n=67%), *KRAS* (52%), *EGFR* (41%), *PIK3CA* (33%), FBXW7 (27%), *PTEN* (24%), *CTNNB1* (22%), *NTRK1* (14%), *TSC1* (14%), *HRAS* (13%), *ERBB2* (12%), *KIT* (11%), *MAP2K1* (11%), *RET* (10%), *BRAF* (9%), *TSC2* (9%), *NTRK3* (7%), *SMAD4* (6%), and *NRAS* (5%). **Figure 2D** showed the mutually exclusive or co-occurring set of genes. **Figure 3** showed the distribution of mutations in *TP53, APC* and *KRAS* protein. The high frequency hotspots of *TP53* (**Figure 3A**) were gathered in *p.G113S/D/C* (n=18) and *p.R43H/C* (n=11). While hotspots of *APC* (**Figure 3B**) and *EGFR* (**Figure 3C**) were relatively dispersed.

### 3.3 Genomic features of CRC anatomic location

**Figure 4:**
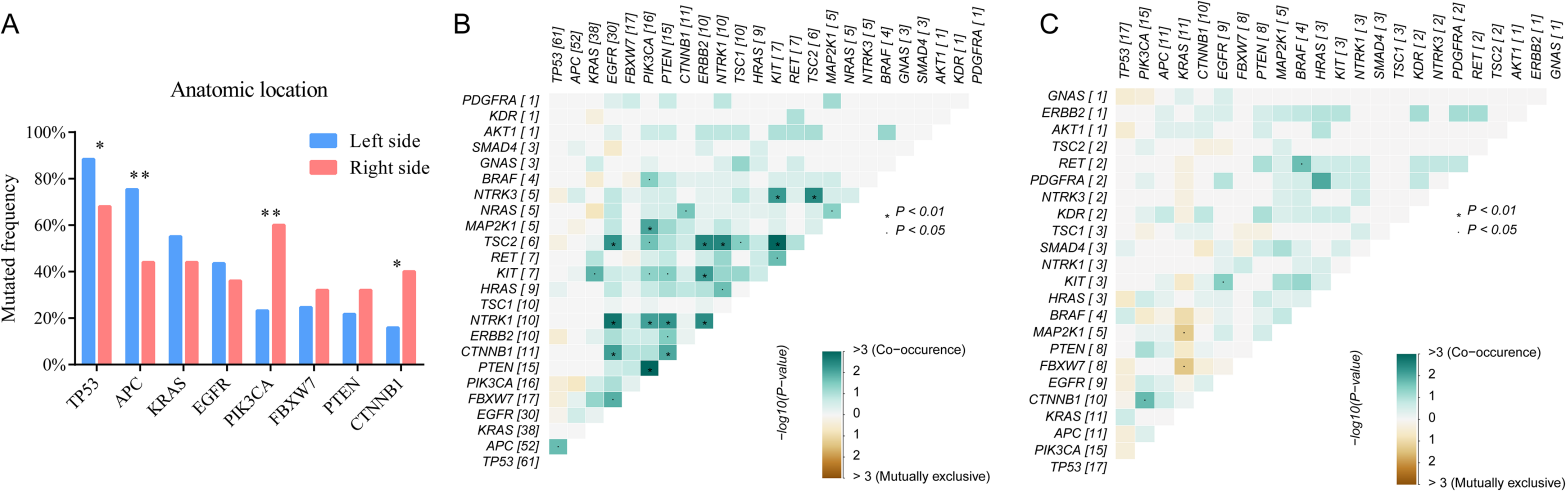
The comparisons of somatic mutated genes among patients with different tumor anatomic locations **(A)** and genetic interaction analysis of left-sided **(B)** and right-sided **(C)** tumors.

According to anatomic location, 94 colorectal tumors were classified as left sided tumors (n=69) and right sided tumors (n=35). No significant correlation was found between tumor location and the clinical characteristics (**Table 1**). We found the genomic features between left sided and right sided tumors were distinctly different (**Figure 4**). The mutated frequency of *TP53* (88.4% vs 68.0%, *P*=0.02) and *APC* (75.4% vs 44.0%, *P*=0.004) in left sided tumors was significantly higher than that of right sided tumors. However, mutated frequency of *PIK3CA* (23.2% vs 60.0%, *P*=0.001) and *CTNNB1* (15.9% vs 40.0%, *P*=0.013) in left side was obviously lower than that of tumors in right side (**Figure 4A**). A big set of mutually exclusive genes were identified in left side tumors (**Figure 4B**), while no co-occurring mutated genes were found. In right tumors, *KRAS* mutated concurrently with *MAP2K1* and *FBXW7*, while only 2 paired of genes showed exclusive mutations (**Figure 4C**).

### 3.4 Tumor features’ impact on PFS

During the follow-up period, 29 patients experienced disease progression (the PFS endpoint), and 11 death events occurred. PFS outcomes were analyzed using Kaplan-Meier curves, and differences between subgroups were compared with the log-rank test (**Supplementary Figure 1**). As expected, tumor stage showed a significant impact, with stages I-II patients having better PFS than stage III (*P*=0.0318). However, tumor location (*P*=0.2786), microsatellite status (*P*=0.1349), mutations in *TP53* (*P*=0.1616), *KRAS* (*P*=0.6318), *EGFR* (*P*=0.7969), *PIK3CA* (*P*=0.6535) and tumor mutation burden (TMB, *P*=0.6922) did not show significant associations with PFS in this cohort. Although not reaching strict significance (*P*=0.0586), *APC* mutated patients had poorer prognosis.

### 3.5 Correlation between blood metal(loid) element level and tumor features

**Figure 5:**
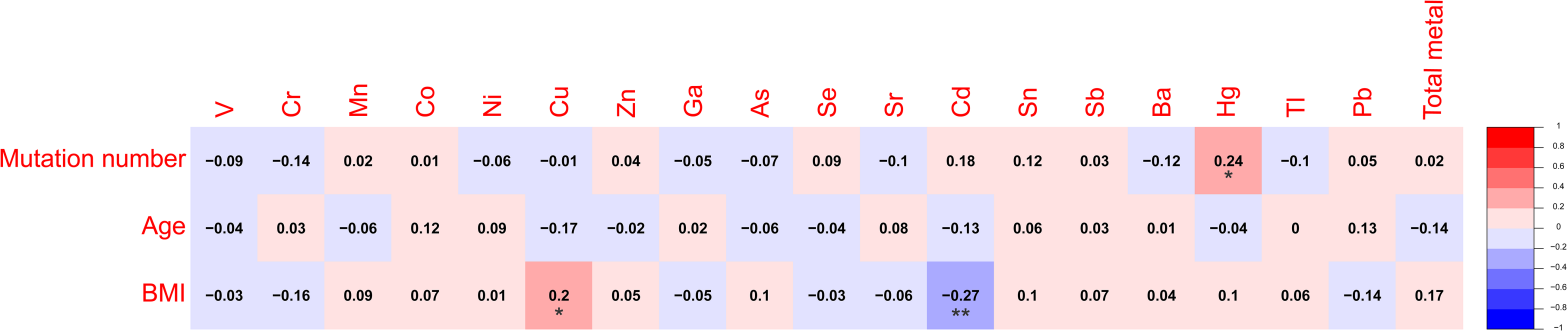
The heatmap showing the correlation among metal(loid) elements’ level in blood, variation number per tumor and patients’ age.

**Figure 6:**
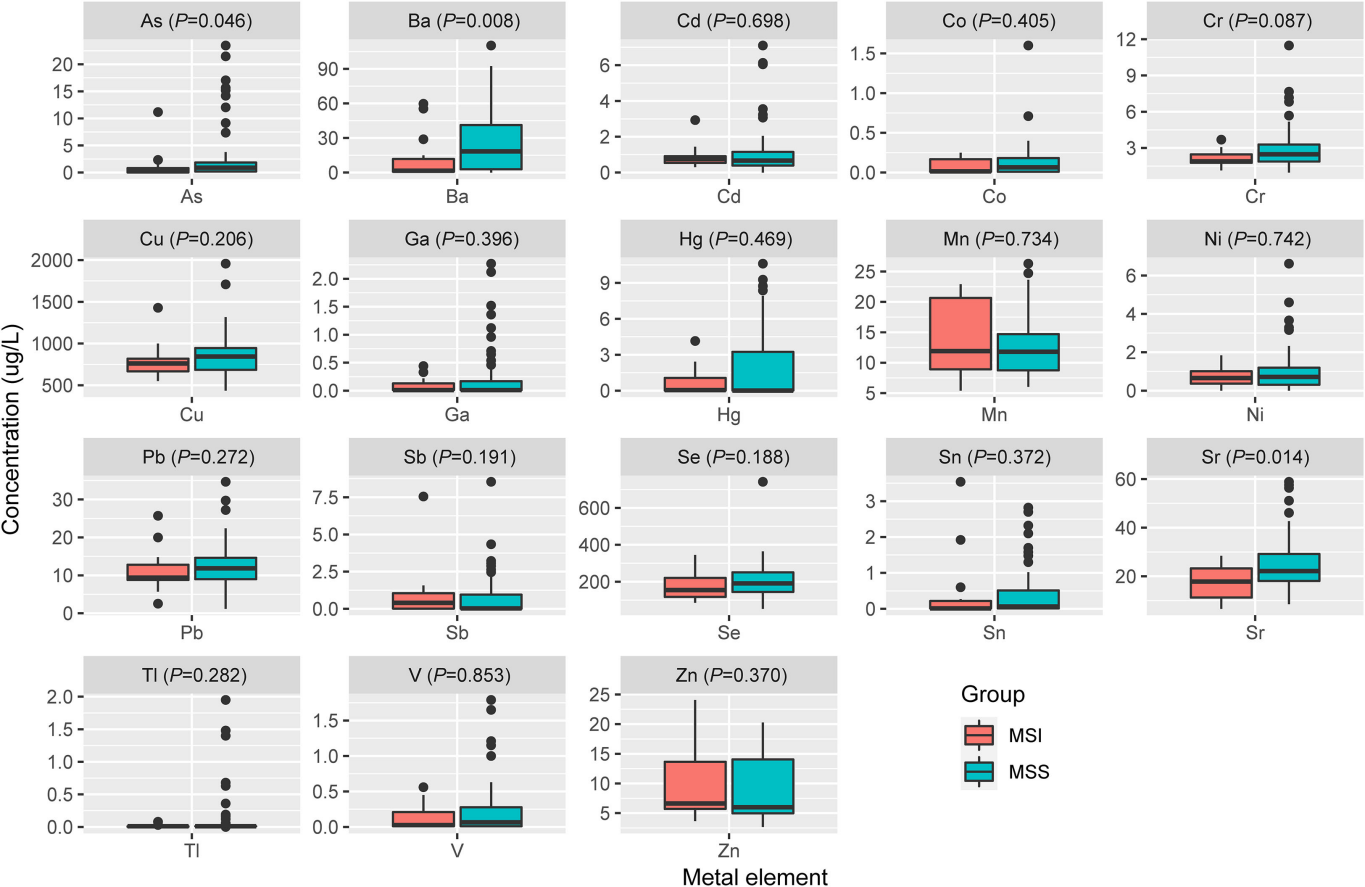
The comparison of metal(loid) elements’ level in blood between patients with MSS tumor and MSI tumor.

**Figure 7:**
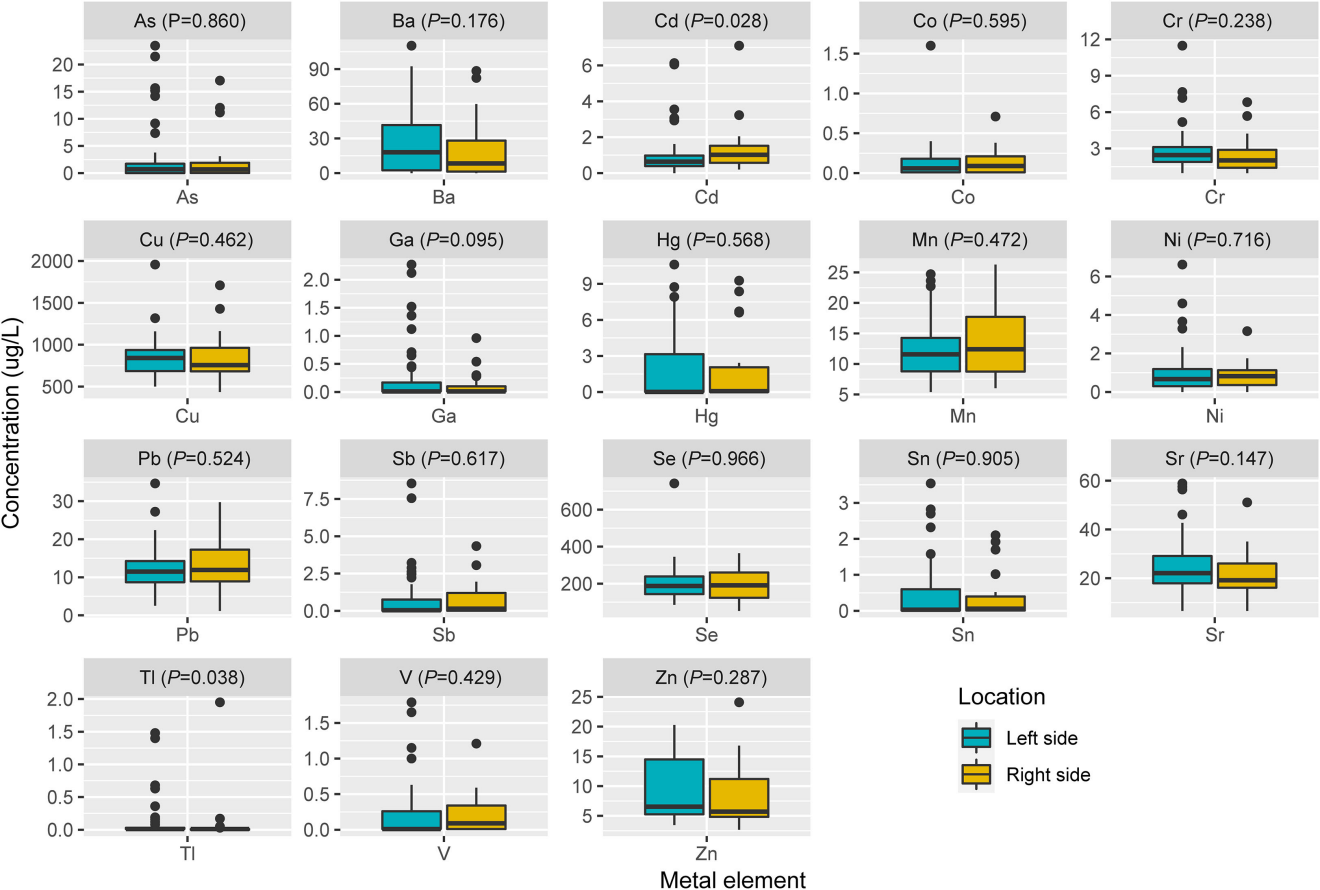
The comparison of metal(loid) elements’ level in blood between patients with left-sided tumor and right-sided tumor.

Spearman’s rank correlation coefficient was calculated between blood metal element level and continuous variables such as mutation number of tumor, patients’ age and BMI (**Figure 5**). Blood Hg concentration was significantly and positively correlated with numbers of variations per tumor sample (*r*=0.24, *P*=0.021). Blood As (*P*=0.046), Ba (*P*=0.08) and Sr (*P*=0.014) level of patients with MSS tumor was significantly higher than that of patients with MSI tumor (**Figure 6**). Analysis for MSI-L and MSI-H subgroups showed the As and Ba level was significantly lower that that of patients with MSS tumor (**Supplementary Figure 2**). According to tumor location, we found Cd level of patients with tumor in left side was significantly lower than that in right side (*P*=0.028, **Figure 7**). The top 5 genes with the highest mutation frequencies were selected, and the metal(loid) element levels were compared between patients with mutated and wild-type genes (**Supplementary Figure 3**). We found V level was significant lower in *EGFR* mutated patients (**Supplementary Figure 3D**), than that of *EGFR* wildtype cases. Besides, Pb level was significantly higher in *PIK3CA* mutated patients than that of cases with *PIK3CA* wild type (**Supplementary Figure 3E**).

## 4 Discussion

This study provides a comprehensive analysis of the genetic mutations, MSI, and toxic metal(loid) accumulation in relation to the side-specific distribution of colorectal tumors. Our analysis of tumor mutation profiles revealed that *TP53* and *APC* emerged as the most frequently mutated genes across all CRC samples, with notable differences in their prevalence between left- and right-sided tumors. It generally aligned with established literature on the genetic underpinnings of CRC, but with some especial features. *APC* has been reported as a gatekeeper gene with highest mutated frequency in human colorectal cancer by majority of researchers (16–19), while a portion of literatures (20, 21) reported *TP53* as most recurrently mutated gene. The variation in reported mutation frequencies might be attributed to several factors, including differences in study populations, methodologies, and tumor subtypes. The mutation spectrum can also vary depending on geographic and ethnic factors, as well as the stage of cancer being studied. Besides, the *APC* gene mutation showed a trend (*P*=0.0586) towards being associated with PFS, although it did not reach strict statistical significance. The observed trend toward reduced PFS in *APC*-mutated tumors aligns with prior studies suggesting that *APC* truncating mutations may promote tumor aggressiveness through dysregulation of Wnt/β-catenin signaling, a pathway central to colorectal carcinogenesis (22).

Specifically, the significantly higher mutation rates of *TP53* (88.4% vs. 68.0%, *P*=0.02) and *APC* (75.4% vs. 44.0%, *P*=0.004) in left-sided CRCs suggest a unique genomic landscape that may underlie their distinct clinical behavior and response to therapy. Y. Takahashi et al. have reported the similar phenomena that left-sided CRCs tend to harbor mutations in *TP53* (10). While the exact frequency of *PIK3CA* mutations in left-sided and right-sided CRC may vary across studies, the overall prevalence of *PIK3CA* mutations in CRC tends to be lower in left-sided compared to right-sided tumors (17, 18). It was consistent with the finding of our study. The higher prevalence of *PIK3CA* mutations in right-sided CRC may reflect the distinct molecular landscape of these tumors, which is characterized by a higher degree of MSI. Previous research has shown that DNA mismatch repair pathways frequently occurred in colon tumors located on the right side (23). This differential mutation frequency supports the hypothesis that left-sided and right-sided colorectal cancers have distinct molecular profiles.

Heavy metals, such as mercury (Hg), arsenic (As), lead (Pb), and cadmium (Cd), are well-documented environmental toxins that can adversely affect human health. Chronic exposure to these metals has been linked to various health issues, including cancer. For example, arsenic and cadmium have been classified as carcinogens by the International Agency for Research on Cancer (IARC), with significant evidence supporting their role in increasing cancer risk. Masoudreza Sohrabi has reported that Zn, Cr, Cu, Al, and Pb in cancerous tissues of CRC was significantly higher than that of healthy tissues (24), which indicated that heavy metal played a role in developing CRC. Mengyuan Liu et al’s study reported that Pb, As, and Cd were the most significant contributors to the increased mutation rates (8), which suggested that heavy metal exposure can impact genomic stability in cancer-related genes.

In our study, we found the patients’ blood Hg concentration was positively and significantly correlated with mutation number per tumor sample (**Figure 5**, *r*=0.24, *P*=0.021). Hg is a known environmental contaminant. The exclusive correlation between Hg level and tumor mutation burden observed in this study may be attributed to Hg’s unique genotoxic properties, including its capacity to induce persistent oxidative DNA damage via reactive oxygen species (ROS) (25) and impair DNA repair pathways (26). Unlike other metals analyzed, Hg’s prolonged biological half-life and systemic bioaccumulation likely amplify its mutagenic impact (27). These findings underscored the importance of evaluating metal-specific mechanisms in genomic instability and highlighted Hg as a critical environmental risk factor for mutation-driven carcinogenesis. Cd has been reported to promote CRC metastasis through *EGFR/Akt/mTOR* signaling cascade and dynamics (28). In this study, we found Cd level was significantly higher in right-sided tumors than that in left side (**Figure 7**, *P*=0.028). CRCs exhibit differences in incidence, pathogenesis, molecular pathways, and outcome according to the location of the tumor (29, 30). Previous studies have demonstrated that right-sided CRC had a significantly worse prognosis than left-sided tumors (31–33). We also observed significantly lower blood V levels in CRC patients with *EGFR* mutations compared to those with wild-type *EGFR*.

In this study, we observed significantly elevated blood Pb levels in patients harboring *PIK3CA* mutations compared to those with wild-type *PIK3CA*. *PIK3CA*, a critical regulator of the *PI3K/AKT/mTOR* signaling cascade, is frequently mutated in CRC. However, their association with heavy metal exposure remains underexplored. Pb, classified as a Group 2A carcinogen by the IARC, may exert its carcinogenic effects through multifaceted mechanisms, such as oxidative stress-induced DNA damage via reactive oxygen species (ROS), impairment of DNA replication and repair processes, dysregulation of cell cycle checkpoints, and epigenetic or transcriptional modulation of oncogenes and tumor suppressors (34). Y. Li et al. demonstrated that CRC patients exhibit significantly higher blood Pb concentrations than healthy controls (35), suggesting Pb’s role in CRC pathogenesis. Notably, analogous interactions have been observed in other malignancies; for example, elevated Pb levels correlate with increased ovarian cancer risk in *BRCA1* mutation carriers, implicating Pb in exacerbating genetic susceptibility (36). Our findings highlight a novel correlation between *PIK3CA* mutations and Pb exposure in CRC, suggesting that environmental heavy metals may interact with oncogenic signaling pathways to shape tumor heterogeneity. This underscores the importance of integrating environmental biomarkers into genomic studies to unravel multifactorial drivers of cancer progression.

The current study, while providing valuable insights, is not devoid of limitation that merit discussion. Only blood metal(loid) of CRC patients were tested, without assessing their concentrations within tumor and paired normal tissues. It may not fully represent local tissue exposure, and difficult to infer the impact of the tumor microenvironment. As a result, our findings emphasized the association between systemic toxic metal(loid) exposure and genomic alteration in colorectal cancer, rather than the specific role of metal(loid) within the tumor microenvironment. Future studies with prospective collection and analysis of both blood, tumor and matched normal tissue samples are needed to better elucidate the complex interactions between toxic metal(loid) and tumor microenvironmental dynamics.

## 5 Conclusions

The findings presented in this study offer valuable insights into the molecular heterogeneity and environmental factors contributing to the distinct biological characteristics of left-sided and right-sided CRCs.

## Data Availability

The datasets presented in this study can be found in online repositories. The names of the repository/repositories and accession number(s) can be found below: https://www.ncbi.nlm.nih.gov/, PRJNA1153631.

## Supplementary material

Supplementary Figure 1 Kaplan-Meier progression-free survival curves for patients with CRC according to stage **(A)**, location **(B)**, MSS status **(C)**, *TP53* **(D)**, *APC* **(E)**, *KRAS* **(F)**, *EGFR* **(G)**, *PIK3CA* genotype **(H)**, and TMB level **(I)**. Tumors with the number of mutations at the median value or above are defined as TMB high, and those below the median value are defined as TMB low.

Supplementary Figure 2 The comparison of metal(loid) elements’ level in blood among patients with MSS, MSI-L and MSI-H status.

Supplementary Figure 3 The Comparison of metal(loid) elements’ level in blood between patients with mutated *TP53* **(A)**, *APC* **(B)**, *KRAS* **(C)**, *EGFR* **(D)**, *PIK3CA* **(E)** and patients with wildtype.
