# Supplementary figures and images for: Association between genomic features and toxic metal(loid) accumulation in left-sided and right-sided colorectal cancer
Source: Front Oncol. 2025 Jul 4;15:1584424. doi: 10.3389/fonc.2025.1584424 (PMC12270859; doi:10.3389/fonc.2025.1584424)

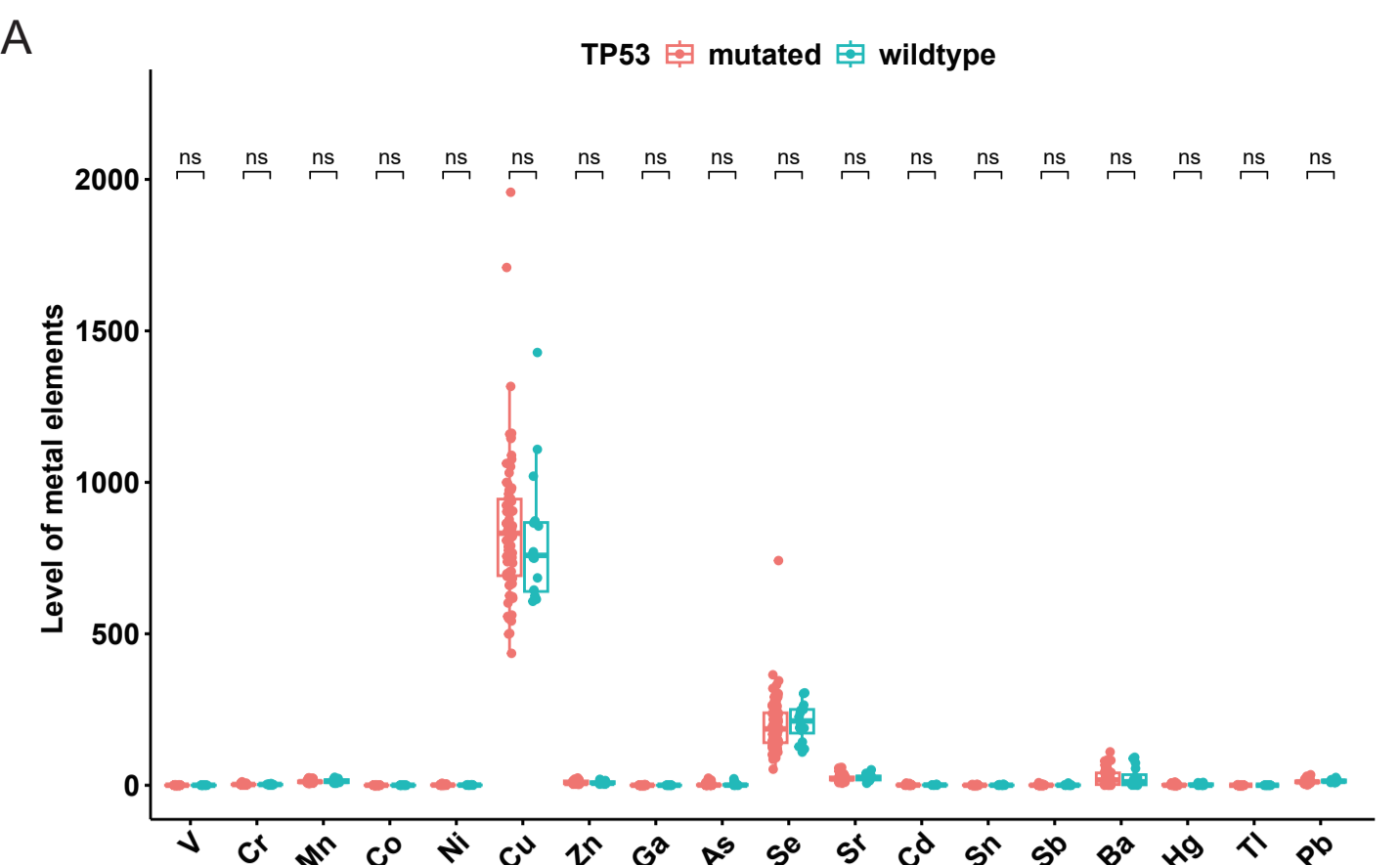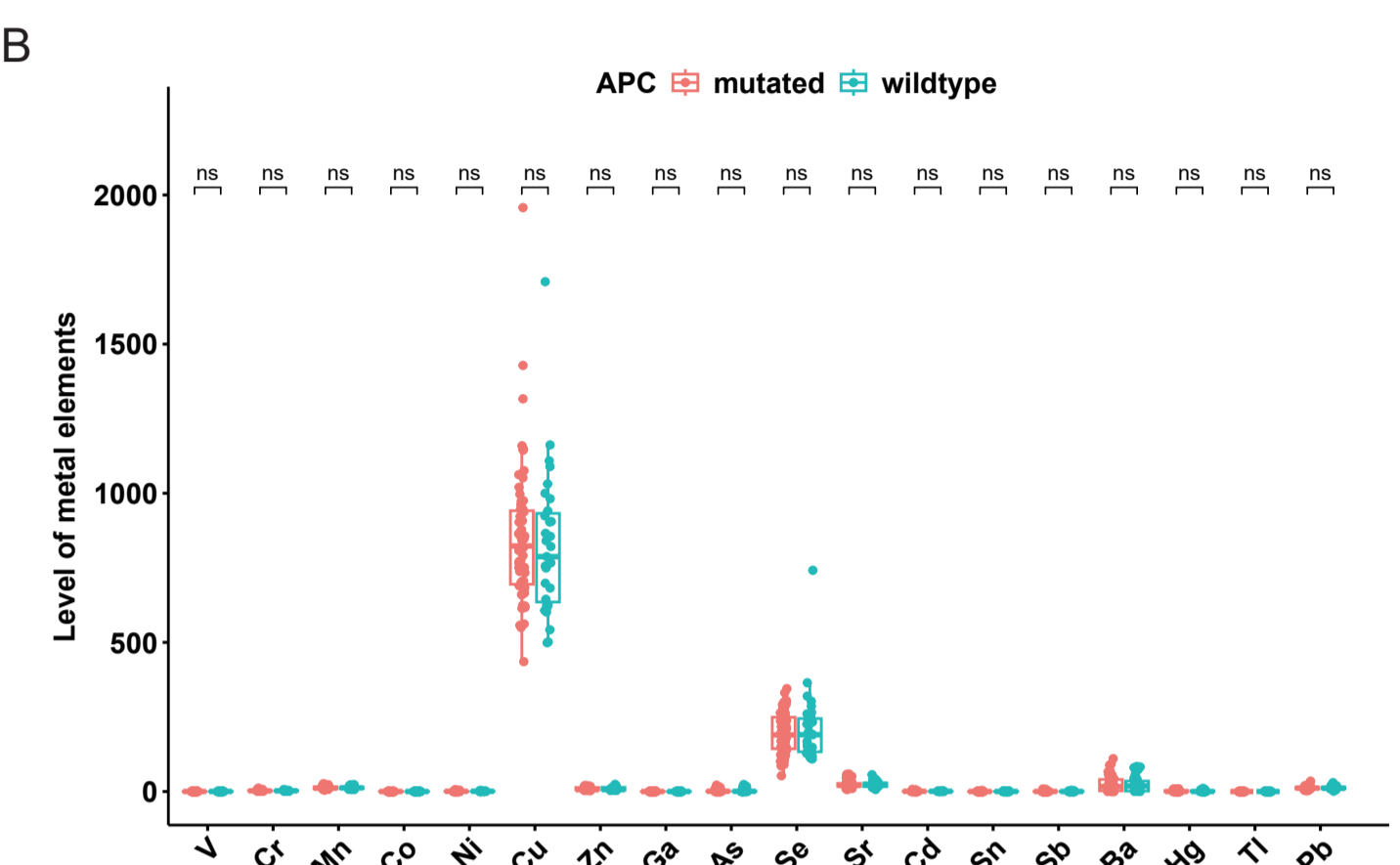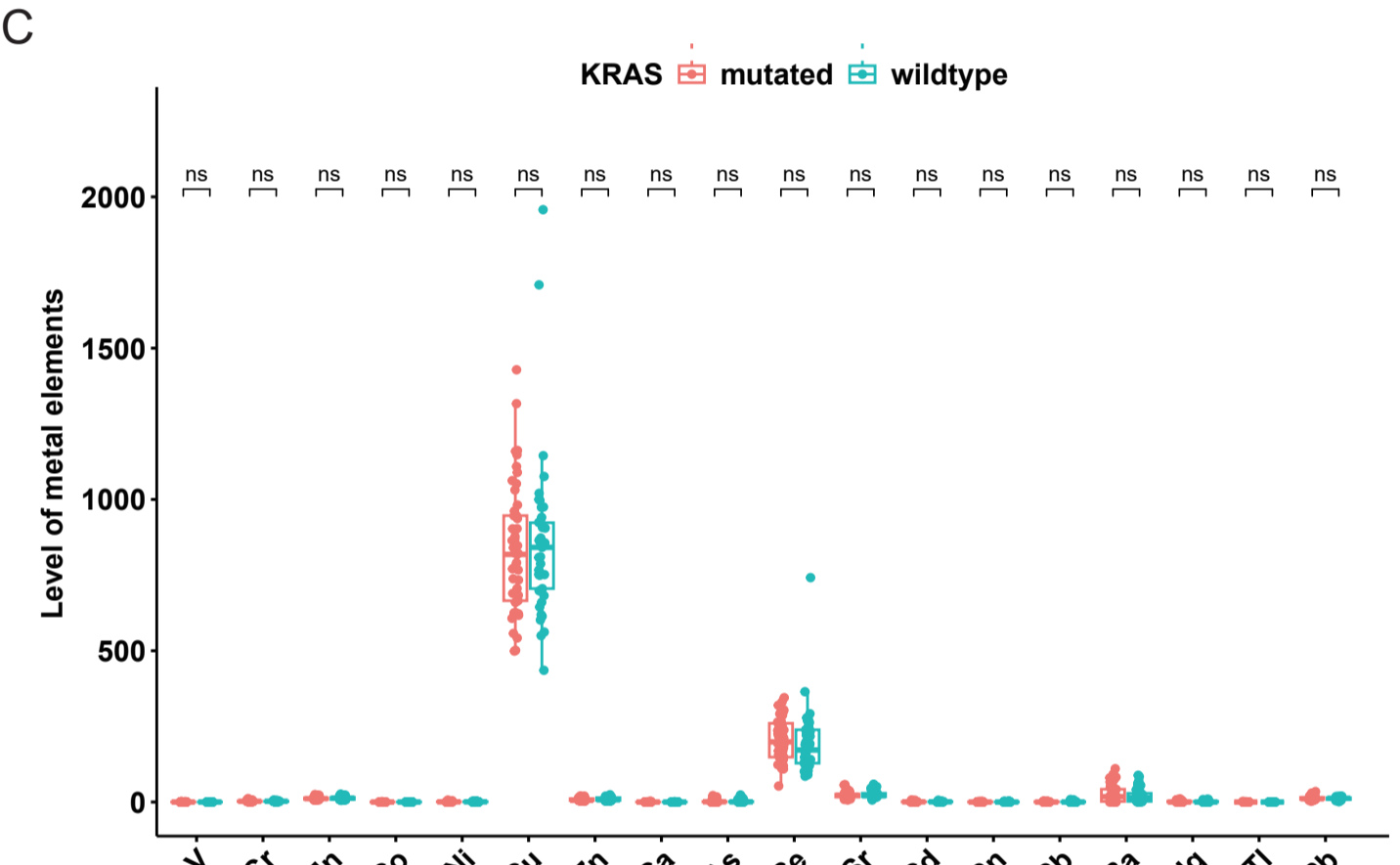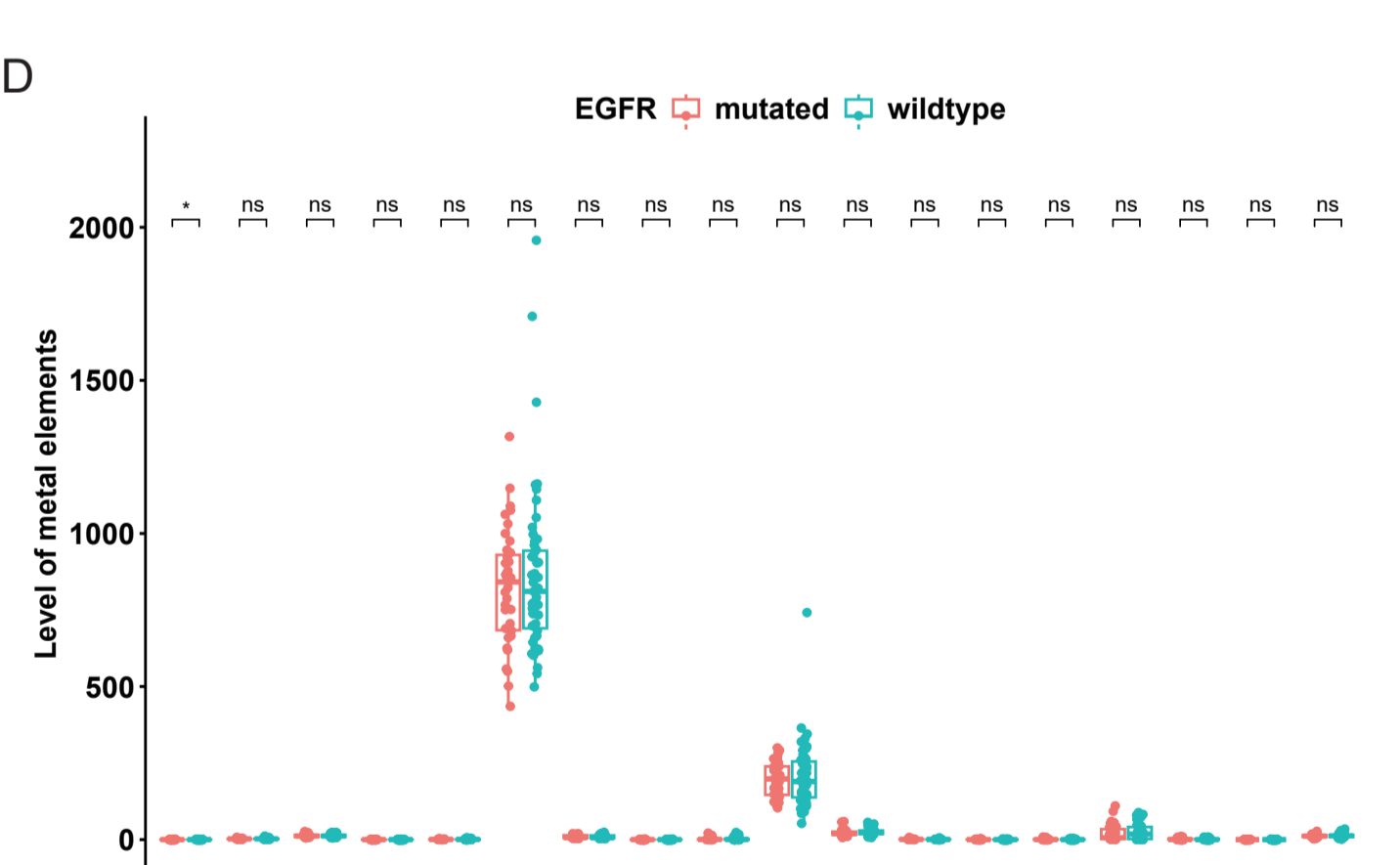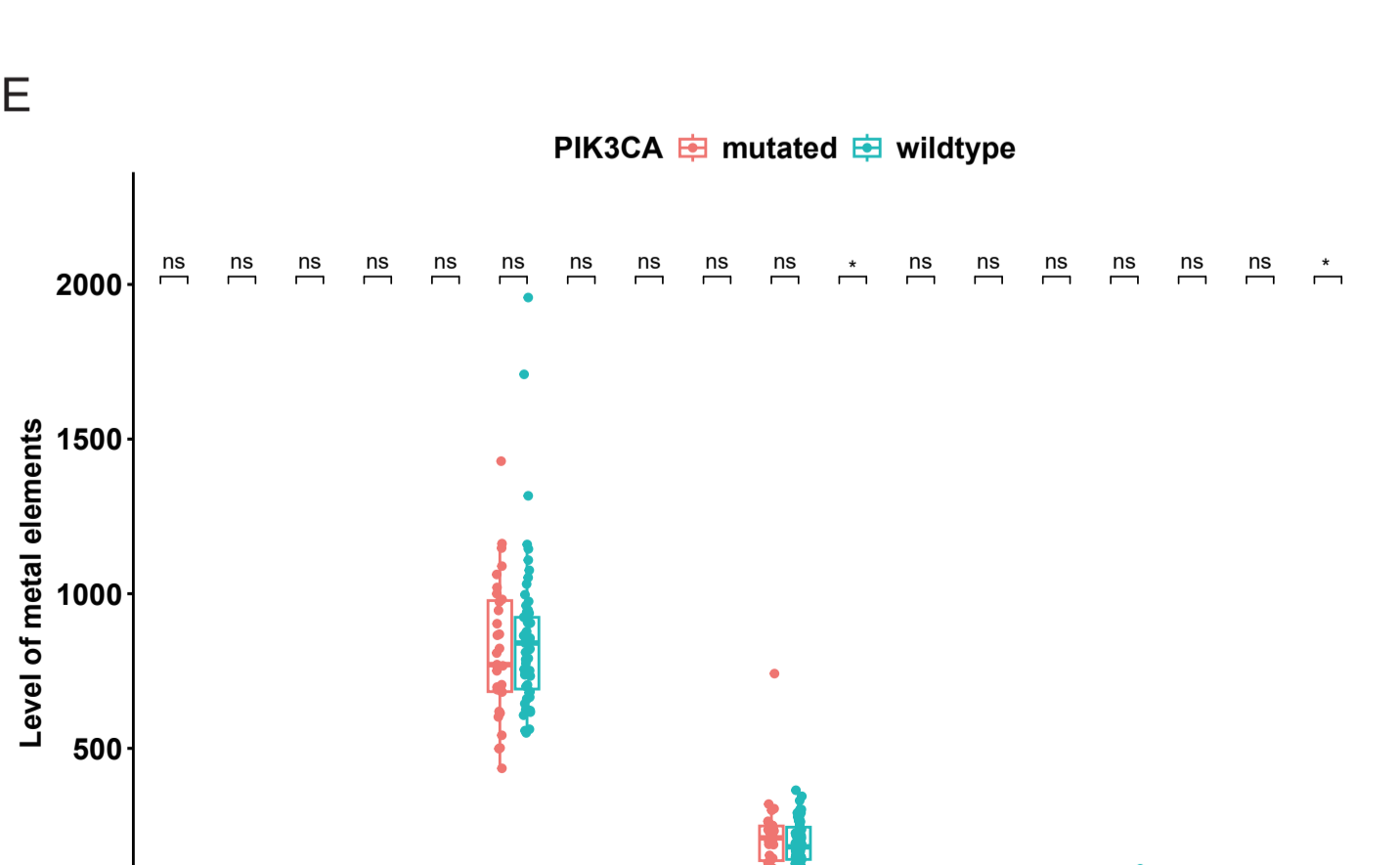

Supplement: Supplementary Figure 3 — The Comparison of metal(loid) elements’ level in blood between patients with mutated TP53 (A), APC (B), KRAS (C), EGFR (D), PIK3CA (E) and patients with wildtype. [file Image3.pdf]
